# A Single Bout of Fasting (24 h) Reduces Basal Cytokine Expression and Minimally Impacts the Sterile Inflammatory Response in the White Adipose Tissue of Normal Weight F344 Rats

**DOI:** 10.1155/2016/1698071

**Published:** 2016-12-18

**Authors:** Kristin J. Speaker, Madeline M. Paton, Stewart S. Cox, Monika Fleshner

**Affiliations:** ^1^Department of Integrative Physiology, University of Colorado, Boulder, CO, USA; ^2^Center for Neuroscience, University of Colorado, Boulder, CO, USA

## Abstract

Sterile inflammation occurs when inflammatory proteins are increased in blood and tissues by nonpathogenic states and is a double-edged sword depending on its cause (stress, injury, or disease), duration (transient versus chronic), and inflammatory milieu. Short-term fasting can exert a host of health benefits through unknown mechanisms. The following experiment tested if a 24 h fast would modulate basal and stress-evoked sterile inflammation in plasma and adipose. Adult male F344 rats were either randomized to* ad libitum* access to food or fasted for 24 h prior to 0 (control), 10, or 100, 1.5 mA-5 s intermittent, inescapable tail shocks (IS). Glucose, nonesterified free fatty acids (NEFAs), insulin, leptin, and corticosterone were measured in plasma and tumor necrosis factor- (TNF-) *α*, interleukin- (IL-) 1*β*, IL-6, and IL-10 in plasma, and subcutaneous, intraperitoneal, and visceral compartments of white adipose tissue (WAT). In control rats, a 24 h fast reduced all measured basal cytokines in plasma and visceral WAT, IL-1*β* and IL-6 in subcutaneous WAT, and IL-6 in intraperitoneal WAT. In stressed rats (IS), fasting reduced visceral WAT TNF-*α*, subcutaneous WAT IL-1*β*, and plasma insulin and leptin. Short-term fasting may thus prove to be a useful dietary strategy for reducing peripheral inflammatory states associated with visceral obesity and chronic stress.

## 1. Introduction

Mammalian white adipose tissue (WAT) has been traditionally regarded as a passive energy storage organ but advances in adipose research have revealed that WAT functions as a highly active endocrine organ serving physiological functions that range from immunity to the regulation of appetite and reproduction [1]. While certain amounts of WAT are required for survival and health, central or visceral obesity has been linked to the etiology of chronic diseases such as cardiovascular disease, type 2 diabetes, and cancer [2, 3]. A growing body of literature suggests that WAT exerts many of its health- or disease-promoting effects through the acute or chronic expression of local inflammatory proteins that function as signaling molecules between the metabolic, immune, and endocrine systems [2–4]. Research regarding the physiology, regulation, and function of WAT-related inflammatory proteins in healthy and obese states is therefore an important area of investigation.

While obesity contributes to the development of disease through the chronic expression of proinflammatory cytokines [3], recent work from our laboratory demonstrates that proinflammatory cytokine expression also occurs in the WAT of normal weight rats which is mediated, in part, by the acute stress response [4, 5]. Rats exposed to 100 acute tail shocks, for example, demonstrate a 10-fold increase in the expression of interleukin- (IL-) 1*β* in the subcutaneous WAT compartment that does not occur in intraperitoneal (epididymal + retroperitoneal depots) or visceral (omental + mesenteric depots) WAT [4]. These findings substantiate the unique responsiveness of WAT relative to its anatomical location and the necessity of studying this organ from a heterogeneous, multicompartment perspective [2, 6, 7].

Unlike the chronic inflammatory state associated with obesity and disease, the acute inflammatory response to nonpathogenic stressors (i.e., sterile inflammation) helps facilitate innate immunity [8, 9], allocate fuels to the immune system [10], and promote host survival [11]. The downstream effects of the immune response, however, can quickly become detrimental if they are not tightly controlled via negative feedback mechanisms [11]. Cytokine expression within WAT is thus a double-edged sword depending on its cause (acute stress, injury, infection, or disease states such as obesity), duration (transient versus chronic), and the local milieu of pro- versus anti-inflammatory proteins (i.e., inflammtaory status) [12]. Despite its primary biological function as an energy reserve, however, the relationship between fed status and cytokine expression in WAT remains unexplored.

Dietary restriction in the form of intermittent bouts of complete food deprivation (aka fasting) with and without chronic caloric restriction has been shown to exert a host of health benefits associated with chronic inflammatory states [13, 14]. Alternate day fasting, for example, can slow the aging process [15, 16], reduce visceral adiposity [17], improve cognitive function [15, 18] and delay the development of chronic diseases [15, 19]. These health-related benefits may be due to improvements in peripheral inflammatory states as sustained caloric restriction has been shown to downregulate nuclear transcription factor (NF)-*κ*B, a key regulator of proinflammtory cytokine expression [20]. To date, however, nearly all of the fasting literature has focused on physiological adaptations to intermittent bouts of fasting rather than on responses to- and effects of an acute, or single bout of fasting. Without this knowledge, the mechanisms whereby fasting exerts its health-related benefits cannot be fully elucidated.

Given the link between obesity-induced inflammation and the pathogenesis of disease [3], short-term fasting may produce immediate health benefits by negatively regulating systemic and/or WAT-related proinflammatory cytokine expression. On the other hand, because of its profound effect on immunomodulatory hormones such as insulin, leptin, and corticosterone [21–23], fasting may negatively affect the body's ability to generate a sterile inflammatory response that, in turn, could compromise host defense in the face of injury or infection. No studies to date, however, have investigated these questions. Moreover, while previous studies have demonstrated that metabolic responses to acute stressors are highly time and/or intensity sensitive [21, 24], the temporal nature of the sterile inflammatory response in WAT has yet to be explored.

The primary aims of this study were to investigate the impact of a short-term fast (24 h) on basal cytokine expression and the sterile inflammatory response to 10 or 100 acute tail shocks in the plasma and subcutaneous, intraperitoneal, and visceral WAT of normal weight rats. We hypothesized that a 24 h fast would reduce basal and stress-induced cytokine expression. Plasma glucose, nonesterified fatty acids (NEFA), insulin, leptin, and corticosterone were assessed as biomarkers of energy status and tumor necrosis factor- (TNF-) *α*, IL-1*β*, IL-6, and IL-10 were measured to determine inflammatory status.

## 2. Materials and Methods

### 2.1. Animals

Adult, inbred male Fischer 344 rats were purchased from Harlan Laboratories (Denver, CO), housed individually in Nalgene Plexiglas cages (45 × 25.2 × 14.7 cm), and kept in the University of Colorado at Boulder's pathogen-free animal facility. Lights were maintained on a 12 : 12 h light/dark cycle (lights on at 0700 and off at 1900) in a temperature (22°C) and humidity-controlled environment. Rats were 9-10 weeks old (~230 g) upon arrival and acclimatized to the facility for one week prior to the experimental protocol. Rats were fed a 2018 Teklad Global 18% protein diet (Harlan Laboratories Denver, CO) with* ad libitum* access to food and water at all times outside of the fasting period. All experimental protocols were approved by the University of Colorado Animal Care and Use Committee.

### 2.2. Short-Term Fasting Protocol

Using a two-step randomization process, rats were first assigned to either FED (*n* = 24) or FASTED (*n* = 26) conditions and next assigned to 0 (no stress control; *n* = 16), 10 (*n* = 18), or 100 (*n* = 16) inescapable tail shocks (IS). The randomization procedure thus resulted in 6 groups as follows: FED + 0IS (*n* = 8), FED + 10IS (*n* = 8), FED + 100IS (*n* = 8), FASTED + 0IS (*n* = 8), FASTED + 10IS (*n* = 10), and FASTED + 100IS (*n* = 8). At 0800, twenty-four hours prior to tail shock stress, all rats were weighed and the food pellets (2018 Teklad Global 18% protein diet, Harlan Laboratories Denver, CO) carefully removed from the cages of the rats assigned to the FASTED condition. Note, special care was taken to ensure there were no remaining pellets in the bedding.* Ad libitum* access to water was provided for all animals at all times. At 0800 on the day of stress, half of the control rats (FED + 0IS, FASTED + 0IS) were sacrificed while the rats assigned to the stressor conditions were exposed either to 10 or 100IS. To account for the time required to administer 100 tail shocks (~100 minutes), the other half of the control rats were sacrificed at ~1000. All rats were weighed immediately prior to sacrifice (control) or tail shock stress.

### 2.3. Acute Tail Shock Stressor Protocol

Between 0800 and 1100 h on the day of stress, rats assigned to the stressed groups were taken to a separate room where they were placed in plexiglass restraining tubes (23.4 cm long and 7.0 cm in diameter) and exposed to 10 or 100, 5 s, 1.5 mA inescapable tail shocks (IS) delivered at random with an average interval of 60 seconds. Control (no-shock/0IS) FED + 0IS and FASTED + 0IS rats remained in their home cages during the stress procedure. The tail shock model was chosen because the uncontrollable and unpredictable nature of the stressor places a high degree of psychological challenge upon the animal that produces a consistent sterile inflammatory response in stress-sensitive F344 rats [4, 25, 26]. Ten (10IS) and 100IS were chosen to address the temporal and/or intensity-sensitive nature of metabolic responses to acute stress [21, 24].

### 2.4. Plasma and White Adipose Tissue Collection

Immediately following the tail shock procedure rats were rapidly decapitated, blood was collected, and WAT was dissected as previously described [4]. Briefly ~0.35 g each of subcutaneous, epididymal, retroperitoneal, omental, and mesenteric WAT was dissected and immediately spot-frozen in liquid nitrogen. To reduce variability, WAT was taken from the right side of the animal (dissectors left) by the same dissector for all subjects. Full anatomical detail, including dissection pictures, can be found in the supplemental material of Speaker et al. 2014. Trunk blood was collected in EDTA tubes (Greiner Vacuette, Monroe, NC) and spun at 3000 g in a chilled centrifuge (4°C) for 15 min. Plasma was removed and stored in a Legaci™ refrigeration system at −80°C (Kendro Laboratory Products, Asheville, NC) for later analysis. Plasma and WAT samples were stored at −80°C (Kendro Laboratory Products, Asheville, NC) until being processed via homogenization. For the homogenization procedure, tissue samples were added to an ice-cold radioimmunoprecipitation (RIPA) lysis buffer (0.5 M Tris-HCl, pH 7.4, 1.5 M NaCl, 2.5% deoxycholic acid, 10% NP-40, 10 mM EDTA, 1 mM NaF, 1 mM sodium orthovanadate, and 1 mM phenylmethylsulfonylfluoride) containing protease and phosphatase inhibitors [protease inhibitor cocktail tablet (Roche, Indianapolis, IN) and 0.01% phosphatase inhibitor cocktail (Sigma, St. Louis, MO)] as previously described [27]. Immediately following the addition of the buffer WAT samples were homogenized by rapid shaking with ceramic beads (2 × 45 s @ 5000 rpm) using a Precellys 24 high-throughput tissue homogenizer (Bertin Coretroperitoneal USA, Rockville, MD). To minimize variability in total protein concentrations between samples each tissue sample was weighed using a Sartorius R200D digital scale (Bohemia, NY) and the volume of RIPA lysis buffer adjusted to a ratio of 3 mL of buffer per gram of tissue. Following homogenization, tissue lysates were stored at −80°C (Kendro Laboratory Products, Asheville, NC) for later analysis.

### 2.5. Assessment of Protein Concentrations in Plasma and White Adipose Tissue

Plasma glucose levels were measured immediately following decapitation using an ACCU-CHEK Aviva Plus System (Roche Diagnostics Indianapolis, IN) while NEFA values were determined using an enzymatic colorimetric assay (Wako Diagnostics, Richmond VA). Plasma corticosterone levels were measured via EIA (Enzo Life Sciences Farmingdale, NY) as previously described [28]. Plasma insulin and leptin concentrations were measured via ELISA (ALPCO Immunoassays, Salem, NH, and R&D Systems Minneapolis, MN) according to the manufacturer's instructions. TNF-*α*, IL-1*β*, IL-6, and IL-10 cytokine concentrations were measured in plasma and WAT lysates by multiplex ELISA (Searchlight Rat Cytokine Assay, Aushon Biosystems, Billerica, MA) according to manufacturer instructions and as described previously [29]. The sensitivity of the multiplex assay was 6.0 pg/mL for TNF-*α* and 0.8 pg/mL for IL-1*β*, IL-6, and IL-10. Notably, we have previously demonstrated that tissues of rats perfused with saline immediately following stress demonstrate similar stress-evoked cytokine profiles compared to nonperfused rats suggesting that the cytokines measured are not the result of any remaining blood in the tissue sample [4].

To account for differences in total protein concentrations between WAT samples, cytokine protein expression is presented relative to the amount of total protein measured (*μ*g of cytokine/mg of total protein) [4, 30]. Protein concentrations of tissue lysates were determined by BCA colorimetric protein assay (Pierce, Rockford, IL) according the manufacturer's instructions. Data from the epididymal and retroperitoneal WAT depots were averaged to yield the intraperitoneal WAT compartment data whereas data from the omental and mesenteric WAT depots were averaged for the visceral WAT compartment [4].

### 2.6. Statistical Analyses

To determine the effect of fasting under basal/nonstressed conditions, unpaired, two-sided *t*-tests were performed between the FED + 0IS and FASTED + 0IS control rats for each of the independent measures. A two-factor analysis of variance (ANOVA) (fed status × stress) was used to analyze the effect of fasting on glucose, insulin, NEFA, leptin, corticosterone, and cytokine responses to tail shock stress in blood plasma; a three-factor ANOVA (fed status × stress × WAT compartment) was utilized to evaluate the effects of fasting on stress-evoked cytokine expression in WAT. Consistent with previously recommended statistical practice, significant two- or three-way interactions were followed by unconfounded pairwise* post hoc* comparisons and evaluated for significance using the Bonferroni/Dunn correction [5, 31, 32].

**Figure 1:**
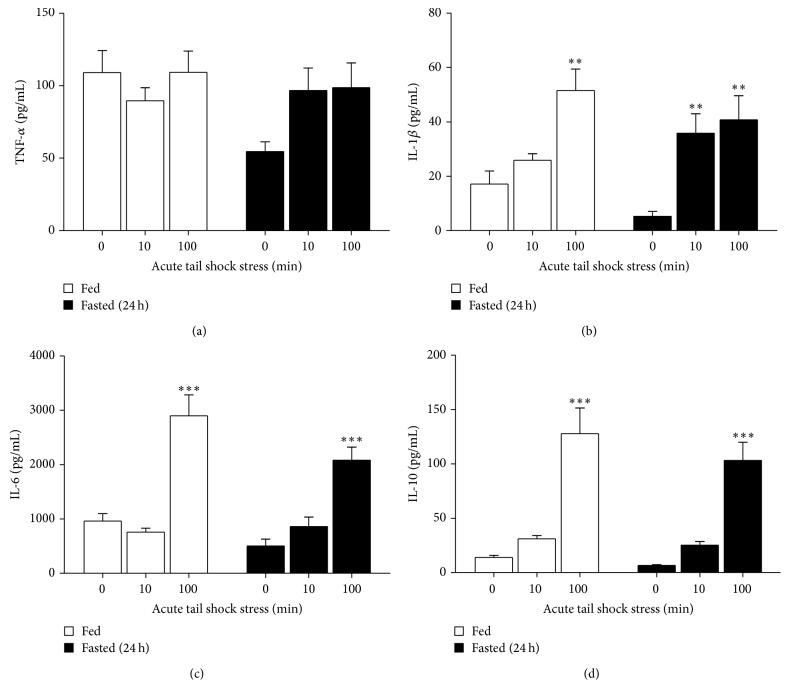
Effect of a 24 h fast on the TNF-*α*, IL-1*β*, IL-6, and IL-10 response to 10 or 100 acute inescapable tail shocks in blood plasma. Fed (FED □) or fasted (FASTED ■) rats were exposed to 0 (control condition), 10, or 100 acute, inescapable tail shocks (IS) and immediately sacrificed and trunk blood was collected. Shown is the mean (+SEM) plasma concentration of (a) tumor necrosis factor alpha (TNF-*α*), (b) interleukin- (IL-) 1 beta (IL-1*β*), (c) IL-6, and (d) IL-10. ^*∗∗*^ *p* < 0.001, ^*∗∗∗*^ *p* < 0.0001 (0IS versus 10 or 100IS).

**Figure 2 fig2:**
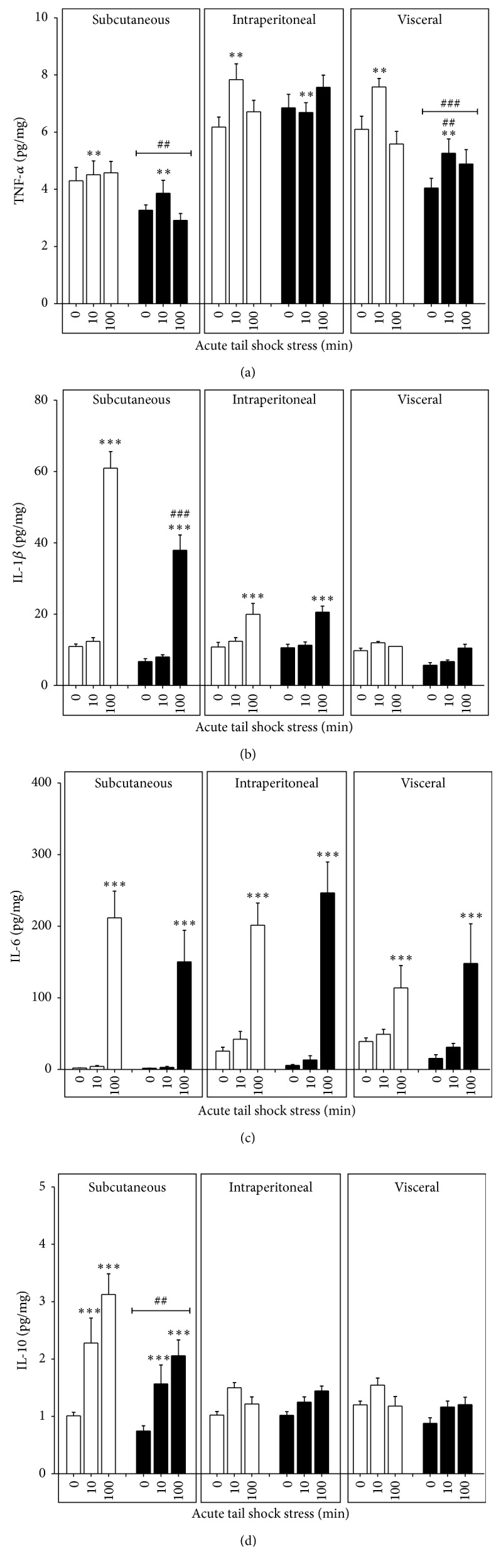
Effect of a 24 h fast on the TNF-*α* and IL-1*β* response to 10 or 100 acute inescapable tail shocks in WAT. Fed (FED □) or fasted (FASTED ■) rats were exposed to 0 (control condition), 10, or 100 acute, inescapable tail shocks (IS) and immediately sacrificed and subcutaneous, intraperitoneal, and visceral WAT were collected. Shown is the mean (+SEM) concentration of (a) tumor necrosis factor alpha (TNF-*α*); ^*∗∗*^ *p* < 0.001 (0IS versus 10IS within WAT compartment), p<0.001##_, p<0.0001###_ (main effect of FED versus FASTED within WAT compartment), and ^##^ *p* < 0.001 (FED + 10IS versus FASTED + 10IS within WAT compartment); (b) interleukin- (IL-) 1 beta (IL-1*β*), ^*∗∗∗*^ *p* < 0.0001 (0IS versus 100IS within WAT compartment and fed condition) and ^###^ *p* < 0.0001 (FED + 100IS versus FASTED + 100IS within WAT compartment); (c) interleukin- (IL-) 6, ^*∗∗∗*^ *p* < 0.0001 (0IS versus 100IS within WAT compartment); (d) IL-10, ^*∗∗∗*^ *p* < 0.0001 (0IS versus 10 or 100IS within WAT compartment), and p<0.001##_ (main effect of FED versus FASTED within WAT compartment).

**Figure 3 fig3:**
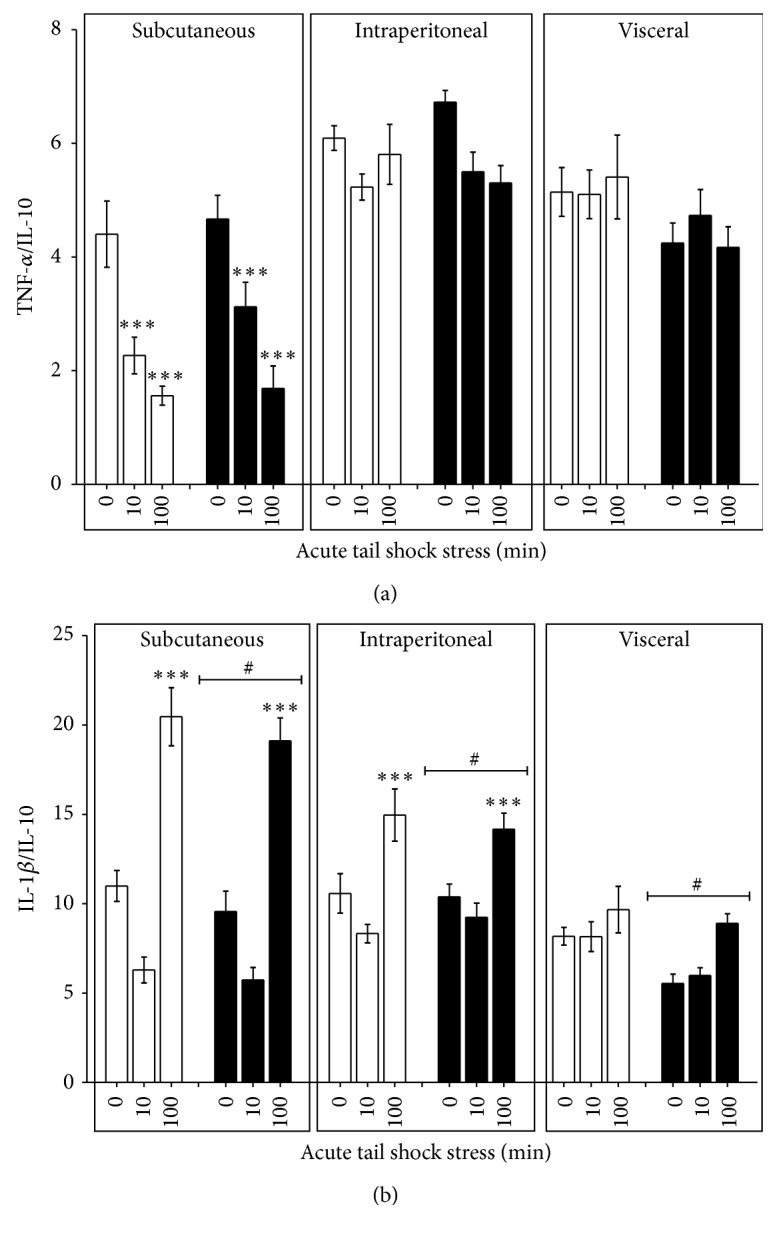
Effect of a 24 h fast on the inflammatory status of WAT following 10 or 100 acute inescapable tail shocks. Fed (FED □) or fasted (FASTED ■) rats were exposed to 0 (control condition), 10, or 100 acute, inescapable tail shocks (IS) and immediately sacrificed and subcutaneous, intraperitoneal, and visceral WAT were collected. Shown is the ratio of the mean (+SEM) concentration of (a) TNF-*α*/IL-10 and (b) IL-1*β*/IL-10, ^*∗∗∗*^ *p* < 0.0001 (0IS versus 10 or 100IS within WAT compartment);  p<0.05#_ (main effect of FED versus FASTED within WAT compartment).

**Figure 4:**
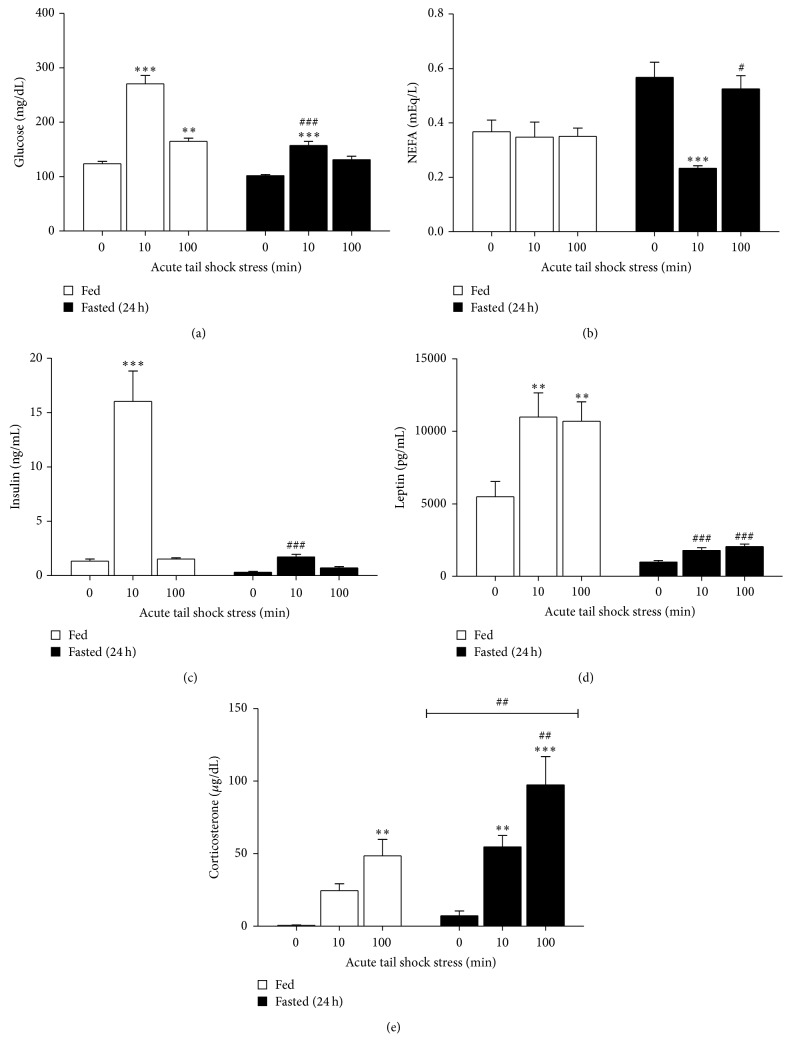
Effect of a 24 h fast on the glucose, NEFA, insulin, leptin, and corticosterone response to 10 or 100 acute inescapable tail shocks in blood plasma. Fed (FED □) or fasted (FASTED ■) rats were exposed to 0 (control condition), 10, or 100 acute, inescapable tail shocks (IS) and immediately sacrificed and trunk blood was collected. Shown is the mean (+SEM) plasma concentration of (a) glucose, (b) nonesterified fatty acids (NEFA), (c) insulin, (d) leptin, and (e) corticosterone. ^*∗∗*^ *p* < 0.001, ^*∗∗∗*^ *p* < 0.0001 (0IS versus 10 or 100IS), p<0.001##_ (main effect of FED versus FASTED), ^#^ *p* < 0.01, ^##^ *p* < 0.001, and ^###^ *p* < 0.0001 (FED versus FASTED within stress condition).

As a secondary* ad hoc* analysis, sample Pearson correlation coefficients between metabolic plasma variables and cytokine expression within each the WAT compartments were assessed for significance using the Fisher transformation. As these were not planned* a priori*, no adjustments were done for multiple comparisons. All data are expressed as means ± standard error of the mean (SEM) and evaluated using Statview software (Statview, SAS Institute Cary, NC). Results were considered statistically significant if *p* < 0.05. Basal and stress-evoked increases hormones and cytokines are depicted in Figures 1 [Fig fig2] [Fig fig3]–4. However, because the stress-evoked levels are so high relative to basal levels, and to better reveal the potential impact of fasting on basal levels of hormones and cytokines, we report basal values and statistics in Tables 1 and 2.

## 3. Results

### 3.1. Body Weight

24 hours prior to tail shock stress, all food was removed from the cages of the rats randomized to the fasted condition (*n* = 26). Mean body weight prior to the onset of food removal was 294 g ± 1.4 for the group assigned to the fed condition and 300 g ± 1.9 for the group assigned to the fasted condition. 24 h later, the mean body weight of the fed rats increased to 294 g ± 1.4 whereas the weights of the fasted rats dropped to 278 g ± 1.9. Having lost approximately 7.0% of their total body weight (−12.9 g), fasted rats weighed significantly less than their fed counterparts (*F*_1,48_ = 45.785, *p* < 0.0001).

### 3.2. Plasma TNF-*α*, IL-1*β*, IL-6, and IL-10

A 24 h fast universally reduced basal cytokine concentrations in blood plasma as shown in Table 1. A main effect of stress was observed for plasma IL-1*β* (*F*_2,44_ = 23.49, *p* < 0.0001), IL-6 (*F*_2,43_ = 42.96, *p* < 0.0001), and IL-10 (*F*_2,43_ = 44.4, *p* < 0.0001).* Post hoc* analyses revealed that 10IS increased IL-1*β* in the fasted group (*p* = 0.0016) while 100IS increased IL-1*β*, IL-6, and IL-10 in both groups (*p* < 0.0001) irrespective of fed status (Figure 1). Note that a plasma sample from one of the 10IS fasted rats was compromised, hence the different *N* for the plasma IL-6 and IL-10 results.

### 3.3. TNF-*α*, IL-1*β*, IL-6, and IL-10 Expression in WAT

As shown in Table 2, a 24 h fast reduced basal IL-1*β* and IL-10 in subcutaneous WAT and IL-6 in intraperitoneal WAT and all visceral WAT cytokines. The effect of fasting on cytokine responses to stress was as follows (Figures 2(a)–2(d)).

#### 3.3.1. TNF-*α*

A significant fed status × WAT compartment interaction (*F*_2,132_ = 7.29, *p* = 0.001) and main effect of stress (*F*_2,132_ = 6.31, *p* = 0.002) were present for TNF-*α*.* Post hoc* analyses revealed that fasting reduced mean TNF-*α* concentrations in subcutaneous (*p* = 0.003) and visceral (*p* < 0.0001) WAT and dampened the TNF-*α* response to 10IS in visceral WAT (*p* = 0.002).

#### 3.3.2. IL-1*β*

A significant fed status × WAT compartment × stress interaction was found for IL-1*β* (*F*_4,132_ = 7.72, *p* < 0.0001).* Post hoc* analyses revealed that fasting prior to 100IS led to lower stress-evoked concentrations of IL-1*β* within the subcutaneous (*p* < 0.001) but not the intraperitoneal (*p* = 0.82) WAT compartment.

#### 3.3.3. IL-6

A significant WAT compartment × stress interaction was found for IL-6 (*F*_4,132_ = 3.73, *p* = 0.007).* Post hoc* analyses revealed that all WAT compartments responded with significant increases in IL-6 protein expression following 100IS (*p* < 0.0001 for all compartments); however, relative to visceral WAT, the intraperitoneal WAT compartment was more responsive (*p* = 0.0001).

#### 3.3.4. IL-10

Significant fed status × WAT compartment (*F*_2,132_ = 4.83, *p* = 0.009) and stress × WAT compartment (*F*_4,132_ = 2.96, *p* < 0.0001) interactions were present for IL-10.* Post hoc* analyses revealed that fasting reduced mean IL-10 concentrations in subcutaneous (*p* = 0.007) WAT while exposure to 10 or 100IS increased IL-10 (*p* < 0.0001), irrespective of fed status.

### 3.4. Inflammatory Status of Subcutaneous, Intraperitoneal, and Visceral WAT

Proinflammatory TNF-*α* and IL-1*β* concentrations were compared in relation to the anti-inflammatory concentration of IL-10 to determine the inflammatory status of each WAT compartment [4, 33]. In the basal (0IS) state, a 24 h fast reduced the IL-1*β*/IL-10 ratio in the visceral WAT compartment (Table 2). Under stressed conditions (Figure 3), a significant stress × WAT compartment interaction was present for both TNF-*α*/IL-10 (*F*_2,132_ = 3.82, *p* = 0.02) (*F*_4,132_ = 7.4, *p* < 0.0001) and IL-1*β*/IL-10 (*F*_4,132_ = 20.68, *p* < 0.0001).* Post hoc* analyses revealed that, irrespective of fed status, exposure to 10 (*p* < 0.0001) and 100IS (*p* < 0.0001) reduced the TNF-*α*/IL-10 ratio in subcutaneous WAT whereas 100IS increased the IL-1*β*/IL-10 ratio in subcutaneous and intraperitoneal WAT (*p* < 0.0001). Fasting reduced the mean IL-1*β*/IL-10 ratio in each of the WAT compartments (*F*_2,132_ = 5.12, *p* = 0.03) but did not affect the 100IS responses.

### 3.5. Plasma Glucose, Nonesterified Fatty Acids, Insulin, Leptin, and Corticosterone

Compared to their fed counterparts, concentrations of glucose, insulin, and leptin were 17%, 76%, and 82% lower, respectively, in the FASTED + 0IS control group whereas plasma NEFAs were increased by 54% along with a strong trend for increased corticosterone levels (Table 1). The impact of a 24 h fast on the metabolic responses to acute tail shock stress was as follows (Figure 4).

#### 3.5.1. Glucose

An interaction between fed status and stress (*F*_2,28_ = 19.17, *p* < 0.0001) was present for plasma glucose.* Post hoc* analyses revealed that fasting significantly dampened the blood glucose response to 10IS (*p* < 0.0001). In response to 100IS, only the fed rats demonstrated significantly higher levels of blood glucose compared to their respective control (*p* = 0.001).

#### 3.5.2. NEFA

An interaction between fed status and stress (*F*_2,44_ = 3.64, *p* = 0.03) was also present for plasma NEFA. Whereas FED + 10IS and FED + 100IS rats demonstrated no change in NEFA concentrations relative to FED + 0IS controls, fasted rats demonstrated a significant reduction in plasma NEFA following 10 (*p* < 0.0001) but not 100IS.* Post hoc* analyses revealed that NEFA concentrations in the FASTED + 100IS group were significantly higher than their fed counterparts (*p* = 0.003).

#### 3.5.3. Insulin

An interaction between fed status and stress (*F*_2,28_ = 19.17, *p* < 0.0001) was observed for plasma insulin.* Post hoc* analyses revealed that fasting attenuated the 12-fold increase in plasma insulin observed in the FED + 10IS group (*p* < 0.0001). Exposure to 100IS did not affect plasma insulin concentrations in either the fed or fasted state.

#### 3.5.4. Leptin

For plasma leptin, an interaction between fed status and stress (*F*_2,44_ = 3.64, *p* = 0.03) was again present.* Post hoc* analyses revealed that the observed increases in leptin following 10 (*p* = 0.0002) and 100IS (*p* = 0.0004) under FED conditions were completely attenuated by fasting (10IS, *p* < 0.0001) (100IS, *p* < 0.0001).

#### 3.5.5. Corticosterone

Main effects of stress (*F*_2,44_ = 23.49, *p* < 0.0001) and fasting (*F*_1,44_ = 3.2, *p* = 0.001) were present for plasma corticosterone.* Post hoc* analyses revealed that 100IS increased plasma corticosterone under both fed (*p* = 0.0016) and fasted (*p* < 0.0001) conditions. 10IS increased corticosterone only in the fasted group (*p* = 0.0011) and fasted rats demonstrated a larger corticosterone response to 100IS relative to FED + 100IS rats (*p* = 0.0013).

### 3.6. Correlational Relationships between Systemic Markers of Energy Status and Cytokine Expression in WAT

To explore relationships between biomarkers of energy status and cytokine expression in WAT, sample Pearson correlation coefficients were examined for relational strength* ad hoc*. All data are presented in Table 3.

## 4. Discussion

The purpose of the present study was to investigate the impact of a 24 h fast on basal cytokine expression and the sterile inflammatory response to acute tail-shock stress in the plasma and subcutaneous, intraperitoneal, and visceral WAT of normal weight rats. Key discoveries include the following: (1) A single bout of fasting for 24 h is sufficient to reduce basal cytokine expression in the blood and WAT; (2) cytokine expression in visceral WAT is more sensitive to fasting-induced energy deprivation than subcutaneous or intraperitoneal WAT; (3) a 24 h fast reduces net concentrations of proinflammatory cytokines in stressed WAT while minimally affecting the nature of the sterile inflammatory response in WAT or blood plasma; and (4) a 24 h fast attenuates stress-evoked insulin and leptin in a time and/or intensity dependent manner.

Corroborating previous reports, a 24 h fast significantly reduced body weight [34] and altered systemic markers of fed status including plasma glucose, NEFA, insulin, and leptin [21, 22, 35, 36]. This study further demonstrates that a 24 h fast is sufficient to reduce cytokine expression in the blood and WAT under nonstressed conditions. Shown in Tables 1 and 2, a 24 h fast reduced basal cytokine concentrations in the plasma by ~50% and 2–60% in WAT depending on the cytokine and WAT compartment. IL-6, IL-1*β*, TNF-*α*, and IL-10 were reduced in the visceral WAT compartment by ~60%, 24%, 34%, and 27% respectively, and inflammatory status (IL-1*β*/IL-10) was lowered by ~32%. Given that elevated levels of proinflammatory cytokine expression in the blood and visceral WAT are the primary drivers of obesity-related diseases [3], these results collectively suggest that a single bout of fasting may aid in health promotion via reducing systemic cytokine release and the proinflammatory state of the visceral WAT compartment. Future studies should examine whether cytokine levels return to baseline following a refeed and, if not, how long a reduction in basal cytokine expression persists and if fasting has similar anti-inflammatory effects under states of chronic inflammation, such as obesity.

This study also demonstrates that, in addition to WAT compartment specificity of the sterile inflammatory response [4, 5], stress-evoked cytokine expression is time and/or intensity dependent and stress induced WAT TNF-*α* and IL-1*β* expression is sensitive to a single bout of fasting (Figures 2(a) and 2(b)). The observed reductions in basal and stress-evoked cytokine concentrations suggest that fasting-induced energy deprivation suppresses cytokine synthesis/release across multiple tissue sites. Changes in the migration of cytokine expressing immune cells from the blood into resident tissues other than WAT [8] and/or alterations in the polarization of infiltrating immune cells within WAT could contribute to these effects [12]. Considering the glucocorticoid antagonist RU486 increases stress-evoked IL-1*β* expression in subcutaneous WAT [5], the dampening of the IL-1*β* response to 100IS observed in the subcutaneous WAT of the fasted rats may be due to an increase in glucocorticoid signaling within the subcutaneous WAT compartment or via reduced inflammasome activation, an essential component to the synthesis of mature IL-1*β* [37]. The observed net reductions in WAT-related TNF-*α* also suggest that fasting may downregulate nuclear factor- (NF-) *κ*B [20, 38], especially in the basal (0IS) state.

Previous work from our lab has shown that rats given access to a running wheel for 6 weeks prior to 100IS demonstrate augmented innate immune function [9] and potentiated sterile inflammatory responses in WAT [4]. Here we present data that fasting dampens stress-evoked TNF-*α* in visceral WAT and IL-1*β* in subcutaneous WAT suggesting a 24 h fast may be detrimental to the sterile inflammatory response. However, because the observed effects of fasting reflect minimal attenuation of stress-evoked TNF-*α* and IL-1*β* in conjunction with a universal reduction in the inflammatory status of WAT (Figure 3(b)), a feature also shown to occur in the WAT of physically active rats exposed to 100IS [4], we speculate that a single bout of fasting is not sufficient to alter the adaptive nature of the sterile inflammatory response. A follow-up study looking at a 24 h fast followed by 100IS and an immediate poststress immunological challenge such as injury or infection is needed to test this hypothesis.

In addition to altering cytokine expression, we discovered a 24 h fast resulted in an unexpected drop in NEFA following 10IS and completely attenuated the insulin and leptin response to 10IS (Figures 4(b), 4(c), and 4(d)). Under conditions of liver glycogen depletion, as is the case after a 24 h fast [13, 39], blood glucose is spared for the brain leaving NEFA as the primary fuel source for peripheral tissues. The observed attenuation of insulin release in addition to a rapid drop in NEFA observed in the fasted rats exposed to 10IS may have thus been due to a need for proper fuel provisioning in the fasted state. In other words, low insulin would reduce peripheral glucose uptake while increases in circulating catecholamines would drive NEFA into metabolically active tissues such as the liver, heart, and skeletal muscle. Fuel partitioning in this manner could also help explain why insulin remained low in the 100IS condition. The dampened increase in plasma glucose and drop in NEFA following 10IS also suggests that, unlike their fed counterparts, fasted rats may have been unable to adequately meet the NEFA demands of acute stressor exposure such that the pituitary-adrenal response (i.e., corticosterone) was further increased to meet the energy demands of stressor exposure. With continued stress exposure fasted rats were able to achieve NEFA homeostasis as reflected by a return to baseline concentrations in the 100IS condition. An additional explanation for the unexpected NEFA data could be that they were confounded by the decapitation procedure [21]. Regardless, these results reveal the importance of considering energy status and the timing of sample collection when investigating endocrine responses to acute stress.

Nakahara et al. reported that leptin responds in a delayed fashion (>60 min) to acute stress [40] but the data presented in this study along with reports from Patterson-Buckendahl et al. suggest that, in the fed state, leptin rises rapidly in response to acute stress and remains elevated throughout stressor exposure [41]. Rats fasted for 24 h, however, failed to demonstrate a leptin response to 10 and/or 100IS (Figure 4(d)). Because compartmentalized leptin stores are filled relative to leptin expression [42], the absence of a stress response suggests that the adipocytes in the fasted rats may have had extremely low levels of intracellular leptin stores as a result of reduced leptin expression. This is important because fasted rats also lacked an increase in leptin after 100IS. As insulin can regulate leptin secretion in a time-dependent fashion (~60 minutes for leptin levels to rise in response to insulin [43]), the rise in leptin observed in the 10IS group may have been due to the release of presynthesized leptin stores, whereas the insulin spike may have activated the synthesis, expression, and release of de novo leptin for the 100IS group [44–46]. Thus the insulin spike observed after 10IS suggests that insulin may have signaled an increase in leptin, which in turn allowed the fed rats to sustain leptin levels throughout the stress response. On the other hand, the lack of insulin both prior to and in response to 10IS in the fasted rats may have reduced presynthesized leptin stores such that there was little to no leptin available for release in response to 10IS. In spite of its profound effect on stress-evoked insulin and leptin, fasting only dampened stress-evoked cytokine expression. Viewed in conjunction with the metabolic data, it is possible that leptin and/or insulin do not act as direct mediators of the sterile inflammatory response.

In an effort to better explore the relationships between metabolic hormones and cytokine expression in WAT, we examined the correlations between circulating markers of energy status and cytokine expression in each of the WAT compartments (Table 3). Interestingly blood glucose, insulin, and leptin were all strongly correlated to cytokine expression in visceral WAT, whereas leptin was the primary correlate in subcutaneous WAT and corticosterone in intraperitoneal WAT suggesting that cytokine expression in visceral WAT may be regulated by energy status to a greater degree than the other WAT compartments. Thus glucose, insulin, and leptin appear to collectively play a role in the mediation of cytokine expression in visceral WAT. If true, approaches targeting systemic levels of these hormones (such as fasting) may be useful for treating inflammatory states associated with visceral obesity. Future studies investigating the mechanisms whereby metabolic hormones mediate cytokine expression in WAT are warranted.

Several strengths to our experimental approach include the assessment of WAT from a multicompartment perspective, the simultaneous assessment of multiple metabolic and inflammatory markers over three time/intensity points, the use of normal weight rats (WAT is rarely studied outside of obesity), and replication of our previously published findings [4, 5]. Conversely, because time and tail shock number increased in parallel, it is unclear whether the differential effects of stress were the function of time or tail shock number. We know from previous work that the plasma cytokines rise with additional tail shocks in nearly a linear fashion and that, by 1-2 hrs after termination of 100IS, plasma cytokines concentrations are back to basal plasma concentration [25]. In addition, the fact that the intensity (1.5 mA), duration (1 s), and unpredictable nature of each shock were the same argues that the differences observed between the 10 and 100IS groups were due to duration versus intensity, per se.

In conclusion, the nature and complexity of negative regulatory mechanisms and how they contribute toward the coordination of the immune response are only beginning to be understood. This study demonstrates for the first time that a single bout of fasting for 24 h is sufficient to reduce basal cytokine expression in blood and WAT while minimally impacting the overall nature of the sterile inflammatory response. Although these data reveal the acute effects of fasting rather than fasting-induced adaptations, the results suggest that short-term fasting may prove to be a useful dietary strategy for mediating peripheral inflammatory states, especially those associated with visceral obesity and chronic stress.

## Figures and Tables

**Table 1:** Effects of a 24 h fast on basal markers of energy status and cytokine concentrations in blood plasma. Differences between blood plasma variables in fed (FED + 0IS, *n* = 8) versus fasted (FASTED + 0IS, *n* = 8) control rats by unpaired, 2-tailed *t*-test. Values are expressed as means ± SEM.

	FED	FASTED	*p* value	Percent change
Glucose (mg/dL)	**123.34 ± 4.67**	**101.63 ± 2.42**	**0.001**	**−17.6%**
NEFA (mEq/L)	**0.37 ± 0.04**	**0.57 ± 0.06**	**0.01**	**54.1%**
Insulin (ng/mL)	**1.34 ± 0.18**	**0.32 ± 0.07**	**<0.0001**	**−76.1%**
Leptin (pg/mL)	**5491.06 ± 1046.82**	**986.91 ± 114.67**	**0.001**	**−82.0%**
Corticosterone (ug/dL)	0.49 ± 0.35	7.21 ± 3.31	0.06	1369.4%
TNF-*α* (pg/mL)	**102.73 ± 14.64**	**54.56 ± 5.85**	**0.01**	**−46.8%**
IL-1*β* (pg/mL)	**17.16 ± 4.16**	**5.26 ± 1.62**	**0.02**	**−69.3%**
IL-6 (pg/mL)	**962.13 ± 135.52**	**502.61 ± 125.88**	**0.03**	**−47.8%**
IL-10 (pg/mL)	**13.93 ± 1.68**	**6.51 ± 0.75**	**0.001**	**−53.3%**

**Table 2:** Effects of a 24 h fast on basal cytokine concentrations in WAT. Differences between WAT cytokines in fed (FED + 0IS, *n* = 8) versus fasted (FASTED + 0IS, *n* = 8) control rats by unpaired, 2-tailed *t*-test. Values are expressed as means ± SEM.

	FED	FASTED	*p* value	Percent change
Subcutaneous WAT				
TNF-*α* (pg/mg)	4.32 ± 0.47	3.27 ± 0.19	0.06	−24.3%
IL-1*β* (pg/mg)	10.91 ± 0.7	**6.72 ± 0.77**	**0.001**	−**38.5%**
IL-6 (pg/mg)	2.15 ± 0.13	1.9 ± 0.03	0.05	−10.7%
IL-10 (pg/mg)	1.01 ± 0.06	**0.75 ± 0.09**	**0.03**	−**26.7%**
TNF-*α*/IL-10	4.4 ± 0.58	4.67 ± 0.42	0.72	6.1%
IL-1*β*/IL-10	10.99 ± 0.86	9.55 ± 1.15	0.34	−13.0%
Intraperitoneal WAT				
TNF-*α* (pg/mg)	6.18 ± 0.35	6.85 ± 0.48	0.27	10.8%
IL-1*β* (pg/mg)	10.79 ± 1.28	10.46 ± 0.98	0.89	−2.1%
IL-6 (pg/mg)	25.65 ± 5.34	**5.85 ± 1.04**	**0.003**	−**77.1%**
IL-10 (pg/mg)	1.02 ± 0.07	1.02 ± 0.06	0.98	−0.2%
TNF-*α*/IL-10	6.09 ± 0.22	6.73 ± 0.21	0.05	10.3%
IL-1*β*/IL-10	10.57 ± 1.11	10.39 ± 0.71	0.89	−1.8%
Visceral WAT				
TNF-*α* (pg/mg)	6.1 ± 0.46	**4.05 ± 0.33**	**0.003**	−**33.6%**
IL-1*β* (pg/mg)	9.78 ± 0.68	**5.68 ± 0.74**	**0.001**	−**41.8%**
IL-6 (pg/mg)	39.12 ± 5.24	**15.66 ± 5.14**	**0.007**	−**59.9%**
IL-10 (pg/mg)	1.2 ± 0.06	**0.88 ± 0.10**	**0.02**	−**26.7%**
TNF-*α*/IL-10	5.15 ± 0.43	4.45 ± 0.35	0.13	−17.5%
IL-1*β*/IL-10	8.18 ± 0.50	**5.54 ± 0.52**	**0.003**	−**32.2%**

**Table 3:** Correlational relationships between biomarkers of energy status and cytokine expression in WAT. Correlations between blood plasma concentrations and WAT cytokine concentrations for all rats (*N* = 50). Values are expressed as the Pearson correlation constant *r* followed by the (*p* value).

	Glucose (mg/dL)	NEFA (mEq/L)	Insulin (ng/mL)	Leptin (pg/mL)	Corticosterone (ug/dL)
Subcutaneous WAT					
TNF-*α* (pg/mg)	0.28 (0.05)	−0.18 (0.22)	0.24 (0.09)	**0.40 (0.004)**	−0.21 (0.14)
IL-1*β* (pg/mg)	0.01 (0.95)	−0.02 (0.90)	−0.16 (0.26)	**0.37 (0.007)**	**0.35 (0.01)**
IL-6 (pg/mg)	−0.06 (0.70)	−0.02 (0.90)	−0.20 (0.17)	0.26 (0.07)	0.19 (0.18)
IL-10 (pg/mg)	**0.40 (0.005)**	0.22 (0.12)	0.24 (0.01)	**0.49 (0.0002)**	0.20 (0.18)
Intraperitoneal WAT					
TNF-*α* (pg/mg)	0.25 (0.08)	0.02 (0.87)	**0.35 (0.01)**	0.16 (0.28)	0.25 (0.76)
IL-1*β* (pg/mg)	0.02 (0.90)	0.04 (0.81)	−0.12 (0.423)	0.11 (0.46)	**0.46 (0.0007)**
IL-6 (pg/mg)	−0.05 (0.71)	0.01 (0.50)	−0.17 (0.25)	0.17 (0.24)	**0.36 (0.01)**
IL-10 (pg/mg)	**0.40 (0.004)**	−0.19 (0.18)	**0.35 (0.01)**	0.25 (0.09)	**0.30 (0.03)**
Visceral WAT					
TNF-*α* (pg/mg)	**0.58 (<0.0001)**	−0.17 (0.23)	**0.54 (<0.0001)**	**0.48 (0.00003)**	−0.09 (0.53)
IL-1*β* (pg/mg)	0.35 (0.14)	−0.15 (0.31)	**0.33 (0.02)**	**0.46 (0.0007)**	0.07 (0.66)
IL-6 (pg/mg)	−0.02 (0.87)	0.02 (0.88)	−0.08 (0.60)	0.09 (0.53)	0.20 (0.18)
IL-10 (pg/mg)	**0.42 (0.002)**	−0.25 (0.08)	**0.43 (0.002)**	**0.36 (0.01)**	0.10 (0.49)
